# Non-maize hosts outperform maize in sustaining fall armyworm population during off-season irrigation in the tropics

**DOI:** 10.3389/fpls.2026.1768817

**Published:** 2026-02-25

**Authors:** Yohannes Ebabuye Andargie, Mintesnot Worku Bogale, Abaynew Jemal Jenber, Alemu Abate, Dong-Sun Lee, Jae-Ho Shin

**Affiliations:** 1 Department of Applied Biosciences, Kyungpook National University, Daegu, Republic of Korea; 2 Next-Generation Sequencing (NGS) Core Facility, Kyungpook National University, Daegu, Republic of Korea; 3 Department of Plant Sciences, Bahir Dar University, Bahir Dar, Ethiopia; 4 Plant Protection Research Team, Gondar Agricultural Research Center, Amhara Agricultural Research Institute, Bahir Dar, Ethiopia; 5 Bio-Health Materials Core-Facility Center, Jeju National University, Jeju, Republic of Korea

**Keywords:** fall armyworm, green-bridge, host preference, irrigation, pest carry-over

## Abstract

The fall armyworm (FAW), *Spodoptera frugiperda*, has emerged as a major global threat to maize production due to its highly invasive nature, absence of diapause, and capacity for continuous reproduction in tropical environments. While off-season irrigated maize is generally recognized as a “green-bridge” for FAW population continuity between seasons, yet its actual contribution relative to alternative hosts remains unclear. To quantify the roles of maize and non-maize hosts in sustaining FAW populations across seasons under climatic continuity and cropping system structures typical of tropical agroecosystems, we assessed FAW seasonal persistence by integrating controlled cage experiments with intensive pest surveys across 123 fields during both irrigated and rain-fed seasons in the Koga Irrigation Scheme, northwestern Ethiopia. Host preference assays revealed that FAW exhibited little host discrimination, feeding readily on finger millet and barley and causing 100% plant mortality, while wheat exhibited 75% severity by 30 days after larval hatching (DAH). Tef sustained significantly slower damage progression (10%, *P* < 0.001), yet suffered total destruction in the absence of maize. Field survey exhibited near-ubiquitous FAW presence (99.2% prevalence), with 4.3-fold increased FAW incidence and 2.9-fold increased severity (*P* < 0.001) during the irrigation season. Structural equation modeling further showed that potato and finger millet exert the strongest positive effects on FAW incidence (β = 0.73 and β = 0.65, respectively; *p* < 0.001), followed by maize (β = 0.49, *p* < 0.01). Elevated infestations after crop rotations, combined with the minimal host preference, demonstrated that FAW persists throughout the irrigation season regardless of crop type. This makes off-season irrigated fields a critical “green bridge”, with some alternative hosts contributing better than maize in sustaining FAW populations. Integrating intensified off-season FAW management strategies into a coordinated, landscape-level framework would be essential for lowering population carry-over and mitigating pest pressure sustainably in the subsequent cropping seasons.

## Introduction

The fall armyworm (FAW), *Sprodoptera frugiperda* (J E Smith) (Lepidoptera: Noctuidae) is a polyphagous pest that inflicts substantial yield losses in maize farms. For many years since its initial detection, FAW remained restricted to the tropical and subtropical regions of the Americas. However, in 2016, its first invasive outbreak was documented in Africa (Goergen et al., 2016). Shortly after it arrived in the continent’s western hemisphere, it rapidly spread across nearly all of sub-Saharan Africa within months, causing estimated annual maize yield losses of 8.3–20.6 million tons (Day et al., 2017). In subsequent years, invasive populations were reported in the Middle East, Southeast Asia, the Far East, and Australia, heightening global alarm over the pest and underscoring the urgent need for comprehensive management strategies (Sartiami et al., 2020; Heinoun et al., 2021; Sun et al., 2021; Rane et al., 2023).

The intrinsic invasiveness of the FAW could be attributed to its high dispersal ability, large reproductive capacity, absence of diapauses, and wide host ranges (Ashley et al., 1989). Its capacity to develop resistance to pesticides and transgenic crops has enabled it to thrive across diverse cropping systems (Banerjee et al., 2017; Boaventura et al., 2021). Remarkably, the FAW can shorten its life cycle from 90 days to just 30 days in favorable conditions (Capinera, 2002), allowing it to reproduce three-fold faster than in less optimal conditions. Consequently, environmental suitability in the tropics contributes to heightened FAW prevalence and damage, as it promotes continuous pest reproduction without diapause. However, host availability also serves as a key determinant sustaining the FAW’s year-round presence.

In temperate regions, excessively cold winter temperatures disrupt the FAW life cycle. In contrast, in the tropics, the pest maintains its life cycle year-round due to favorable weather and host plant availability. The invasive FAW exhibits greater genetic similarity to the corn strain than the rice strain (Durand et al., 2024). Although these strain names do not necessarily reflect host preferences (Sisay et al., 2024), maize is a well-established primary host for the invasive fall armyworm in the tropics. Consequently, irrigated maize production during the tropical dry season can serve as a key off-season breeding habitat for fall armyworm, serving as a “green bridge” that allows populations to persist and subsequently re-invade main-season maize with higher intensity. Although the role of irrigated maize in sustaining FAW populations is widely recognized, the magnitude of its contribution as a green bridge has not been quantified. Moreover, the contribution of secondary hosts to population carry-over during the irrigation season remains poorly understood, limiting the ability to design crop rotations that effectively reduce infestation risk.

To address these gaps, we conducted extensive pest surveys across 123 maize fields in the Koga Irrigation Scheme (KIS), Ethiopia, during both the main (rain-fed) and irrigation seasons to quantify the influence of irrigated maize and alternative hosts on FAW prevalence. Complementary controlled cage experiments were performed to evaluate host preference and damage progression of *S. frugiperda* among alternative hosts, with the objective of determining whether non-maize crops can sustain FAW populations during the dry season and thereby contribute to seasonal population continuity.

Through our comprehensive investigation, we quantified the prevalence and damage intensity of FAW across both the main (rain-fed) and irrigation cropping seasons. We compared infestation levels between irrigated and rain-fed maize fields and evaluated the role of irrigated maize and rotation crops, serving as potential alternative or secondary hosts, in acting as ‘green bridges’ that sustain pest populations during the dry season. These analyses were conducted using regression models to identify key predictors of FAW incidence and severity. Additionally, we assessed host preferences and damage progression of FAW for common cereal rotation crops grown in the KIS and surrounding regions by simulating larval populations derived from the progeny of two female and one male moth to predict the potential damage inflicted by overwintering adult populations. We also employed structural equation modeling (SEM) to elucidate the complex interrelationships among biotic and abiotic factors driving FAW prevalence, thereby providing an integrative analytical framework to inform evidence-based management strategies and future research priorities.

## Methodology

### Description of the pest survey area

The Koga Irrigation Scheme (KIS) is located in the Upper Blue Nile Basin at 11° 10’and 11°25’ North and 37°2’ and 37°17’ East in *Mecha* district, Amhara National Regional State, Ethiopia. The KIS potentially irrigates a 7000-ha command area using rainwater harvested in a dam from a 22,000-ha catchment in the upper Blue Nile Basin, which drains into the Koga River (Asres, 2016). It is Located approximately 41 km south of Bahir Dar City along the route to Addis Ababa, with an average altitude of 1953 m above sea level. The mean maximum and minimum temperatures are 26.8°C and 9.7°C respectively. Mean annual rainfall, recorded at the station of *Merawi* (the main town of *Mecha* district), was 1480 mm, with 90% occurring from May to October. The dominant soil type in the survey area was Nitisol with strong acidity (pH 5.04 -5.25) and it has medium to high organic matter (2.34% - 4.44%) content. The available phosphorus is moderately good (53–55 *ppm*) while it has medium total nitrogen content (0.2-0.23%) (Asmamaw et al., 2021).

### Sampling, description of variables, data recording parameters

**Figure 1:**
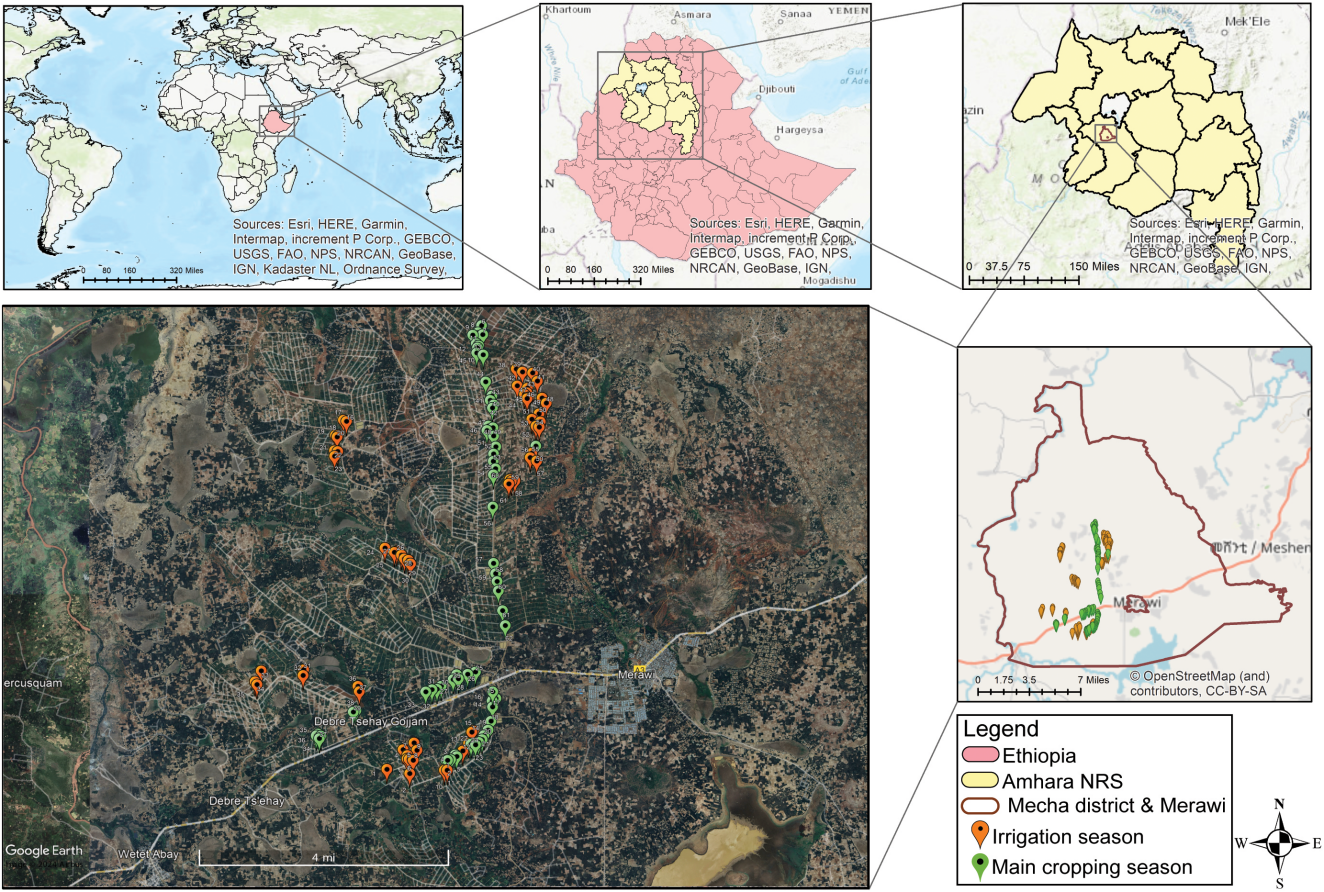
Study area map and sampling points in the main and irrigation season at KIS.

Pest surveys were conducted in maize fields at KIS during the main cropping season in July 2019 and in the irrigation season December, 2019. A total of 123 randomly selected maize fields were surveyed across KIS, including 62 fields during the main season and 61 during the irrigation season (**Figure 1**). The study area encompassed eight irrigation blocks (command areas): Tagel, Chihona, Ambomesk, Tekledib, Bered, Andinet, Amarit, and Lasi. Data were recorded on agronomic and field characteristics, including previous crop, maize growth stage, and weed management as well as pest parameters, such as incidence, and severity expressed as leaf damage percentage (LDP), LDP by damage type (scratching, pinhole, and rugged), and larval instar stages observed on sampled plants. ‘Previous crop’ refers to the crop planted in the specific field during the preceding season. ‘Weed management’ was assessed based on critical weeding periods (Gantoli et al., 2013), and categorized as good, medium, or poor. Fields classified as “good” were weeded twice, once at the 4–6 leaf stage and again at the 8–10 leaf stage, and had an average of <15 weeds m^-^². Fields weeded only once during either period were classified as “medium” and had an average of 65 weeds m^-^². Fields with no weeding until the survey date were classified as “poor” and had >120 weeds m^-^².

The FAW ‘Incidence’ was calculated as the percentage of plants showing FAW infestation relative to the total number of sample plants per field (n=50). FAW severity was recorded based on ‘Leaf damage percentage’ (LDP) referring to the mean percentage of leaves damaged by FAW infestation as indicator of both host preference and host suitability. LDP by damage type was recorded by classifying leaf damage percentage into three FAW-specific categories: scratching, pinhole, and rugged (**Supplementary File 1**: **Supplementary Figure S1**).

In each field (< 2 ha), a total of 50 plants were randomly selected and assessed for FAW incidence and severity. For fields in vegetative stages up to late whorl, plants were sampled using a ‘W’ scouting pattern; for fields in reproductive stages, a ‘ladder’ pattern was used. All 50 sampled plants were individually examined for FAW incidence and severity using the LDP, and larval instars were recorded when live larvae were present.

### Experimental design for host preference and damage progression assessment

The host preference and damage progression of FAW relative to its primary host, maize (*Zea mays* L.), were evaluated on four cereal hosts commonly grown in the region: finger millet (*Eleusine coracana* L. Gaertn.), Wheat (*Triticum aestivum* L.), barley (*Hordeum vulgare* L.), and tef (*Eragrostis tef* [Zucc.] Trotter). The cage experiment was conducted under controlled conditions at the Amhara Agricultural Research Institute, Bahir Dar, Ethiopia following the institutional biosafety guidelines. Each experimental unit consisted of a plastic pot (25 cm top diameter × 15 cm bottom diameter × 20 cm height) filled with a standard soil mixture and planted with one crop species. Immediately after germination, pots were transferred to insect-proof cages (0.75 × 0.75 × 1 m, L × W × H) to prevent external infestation. Host preference tests were conducted using a completely randomized design with three replications per treatment. For each replication, the positions of the cages within the experimental area and the assignment of pots to cages were randomized. In the choice test, four pots—three containing one of the test cereals (finger millet, barley, wheat, or tef) and one containing maize as the preferred reference host were placed together in each cage. In the no-choice test, four pots each containing a single test cereal were arranged within a cage in the absence of maize. All cages were maintained in an open, unshaded area to ensure natural temperature, humidity, and photoperiod conditions.

*S. frugiperda* larvae collected from naturally infested maize fields were reared under laboratory conditions until adult emergence. Newly emerged adults of uniform age were selected for release in the experimental cages. In each cage, two female and one male adult moths were introduced and the cages remained sealed throughout the experimental period to prevent escape or external contamination. Egg masses laid by the released adults were monitored daily until hatching. After hatching, larval feeding damage on each host plant was assessed at 1–3-day intervals for 30 days after hatching (DAH) to evaluate both the damage progress and host feeding preferences (**Supplementary File 1**: **Supplementary Figure S2**).

### Data analysis and statistical modeling

Map of the study area was constructed using ArcMap version 10.7.1. The shape files were constructed using QGIS Desktop version 3.38.0 and the base maps were from the ArcMap 10.7.0 inbuilt sources. Google Earth Pro software was used to display sampling points on the retrieved areal image of *Mecha* district and KIS command areas from Airbus 2024. Descriptive statistical analysis was carried out using SPSS version 25. Parameter estimates of the logistic regression were analyzed with the PROC GENMODE model in SAS version 9.0 to evaluate multiple explanatory variables and comparison across different variable classes. Prior to data analysis, normality test was made using Shapiro-Wilk test and the homogeneity of variances was determined using Levene’s test. Then, statistical tests on differences of FAW incidence and LDP in the main and irrigation season were tested using Mann-Whitney U test and independent Student’s *t*-test, respectively, using R software version 4.4.3. Damage percentage data (**Supplementary Files 2**, **3**) were analyzed using a linear mixed-effects model (LMM) implemented in the lme4 version 1.1-37 (Bates et al., 2015) and lmerTest version 3.1-3 (Kuznetsova et al., 2017) R packages. Model parameters were estimated using restricted maximum likelihood (REML), and the significance of fixed effects was assessed using Type III ANOVA with Kenward–Roger degrees of freedom correction for maize-cereal co-cultivation, while Kruskal-Wallis test followed by Dunns’s test was performed for cereal co-cultivation data. Estimated marginal means (EMMs) for crop damage × DAH combinations were computed using the emmeans package version 2.2.0 (Searle et al., 1980), and pairwise comparisons among host crops at each observation interval were adjusted using the Tukey method to control for multiple testing. Compact letter displays were generated to denote statistically distinct groups. Temporal patterns of damage progression were visualized by plotting EMMs across days for each crop using ggplot2 package version 3.5.1 (Wickham et al., 2016). The SEM was used to assess the direct and covarying effects of experimental factors on fall armyworm incidence and leaf damage percent. Two SEM models were tested: the first evaluated the influence of cropping season, maize growth stage, insect developmental stage, and leaf damage type on incidence and LDP, with both outcomes allowed to covary; the second assessed the effects of previous crops on incidence and LDP. All numeric data were standardized before the analysis, and the SEM correlation matrix was processed using R software version 4.4.3 using lavaan package version 0.6-20 (Rosseel, 2012) and semPlot package version 1.1.6 (Epskamp, 2015).

## Results

### Irrigation season FAW prevalence exceeds the main cropping season

The cumulative prevalence of FAW infestation in both the main and irrigation seasons in KIS maize fields reached 99.2%. Maize was cultivated continuously across seasons in 8.9% of surveyed fields, while 86% were rotated with eight different crop types, dominated by wheat and potato; 4.8% of fields were fallow in the preceding season. Most maize fields were at early to late whorl stages (V3–VT), with 65% infested by 3rd–5th instar larvae. However, 30.9% of fields were dominated by 6th instar larvae. FAW incidence was considerably higher during the irrigation season, ranging from 26% to 96%, compared with 0% to 60% in the main season. This seasonal difference was reflected in the level of crop injury with average leaf damage reached 60% in the irrigation season, whereas it was only 33.6% during the main season (**Table 1**).

**Table 1:** Incidence and Severity of fall armyworm in the main and irrigation cropping seasons at KIS.

Parameters	Irrigation season			Main season			Pooled		
	Mean	(Min-Max)	SD	Mean	(Min-Max)	SD	Mean	(Min-Max)	SD
Incidence	65.4	(26-96)	14.9	23.6	(0-60)	25.1	44.3	(0-96)	12.6
scratching	5.0	(0-48)	10.3	1.9	(0-14)	20.2	3.4	(0-48)	2.9
Pinhole	21.4	(0-78)	12.4	12.3	(0-52)	7.7	16.8	(0-78)	12.7
Rugged	39	(0-86)	20.1	9.5	(0-28)	13.4	24.1	(0-86)	8.3
LDP	60.0	(27.8-98.1)	16.8	33.6	(0-74.1)	21.3	46.7	(0-98.1)	13.7

LDP, Leaf Damage Percentage; (Min-Max), (Minimum – Maximum); SD, Standard deviation

**Figure 2:**
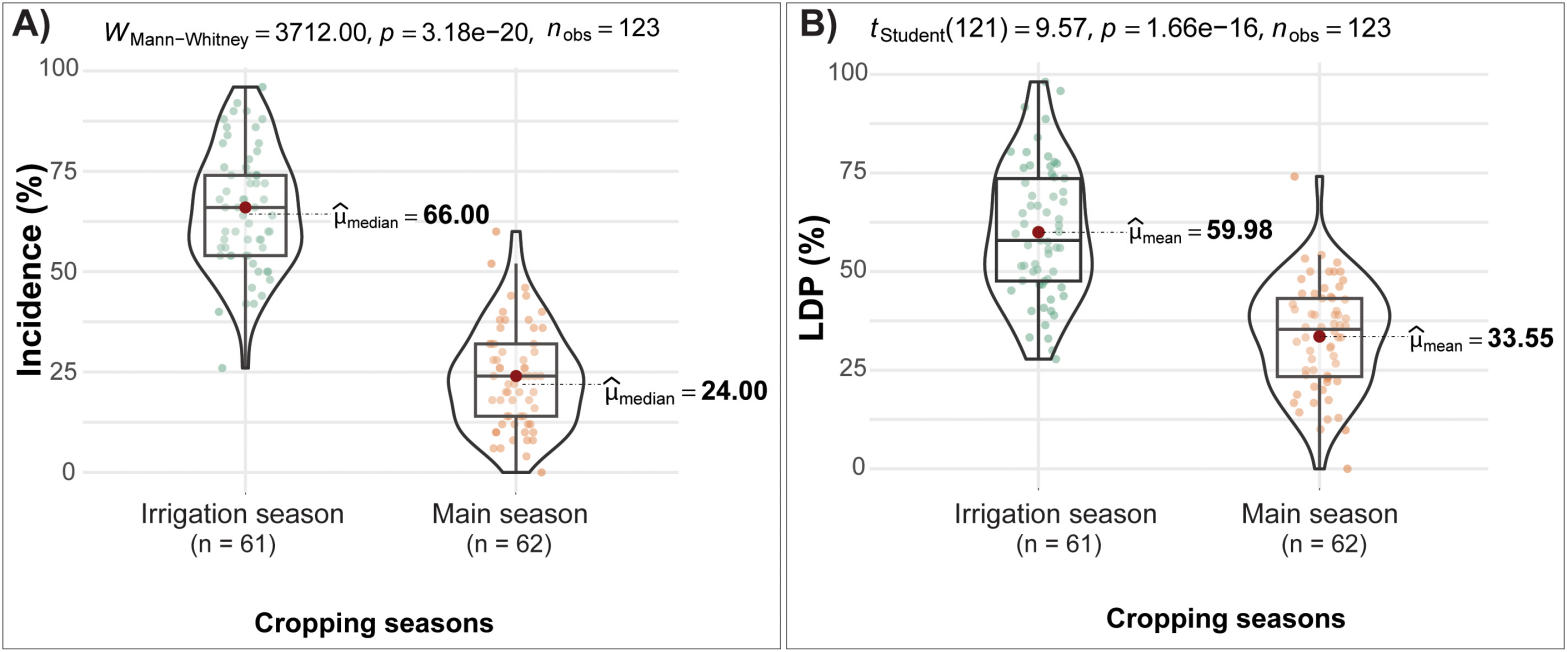
Test of significance for FAW incidence and LDP between maize fields during **(A)** irrigation and **(B)** main cropping seasons.

The Mann-Whitney U test confirmed that FAW incidence in the irrigation season (median = 66%) was significantly higher (*p* < 0.001) than in the main season (median = 24%) (**Figure 2A**).

An independent Student’s *t*-test confirmed significantly higher FAW leaf damage percentage (LDP) during the irrigation season (mean = 59.98%, n = 61) compared with the main season (mean = 33.55%, n = 62; P < 0.001). (**Figure 2B**). Damage type also differed between seasons: rugged damage dominated in the irrigation season (39%), while pinhole damage was more common in the main season (12.3%) (**Table 1**).

### Irrigation and alternative hosts underpin increased FAW incidence and LDP

Logistic regression analysis (**Table 2**) showed that maize fields during the irrigation season had a significantly higher likelihood of fall armyworm (FAW) incidence compared to those in the main cropping season (*OR* = 4.30, *p* < 0.001). Previous crop type also emerged as a significant predictor of FAW incidence in the subsequent season (*p* < 0.01), with distinct risk profiles across preceding crop types (**Table 2**). Fields previously planted with snow pea (*Pisum sativum*), tomato (*Solanum lycopersicum*), and finger millet (*Eleusine coracana*) had a significantly higher risk of FAW incidence (*p* < 0.001) than in onion-preceded fields, exceeding the risk of replanting maize (*Zea mays*) in consecutive seasons (OR = 5.58, 2.77, and 2.58, respectively). Maize-preceded fields were approximately 2.5 times more likely to experience FAW incidence in the following season than onion-preceded fields (OR = 2.48, *p* < 0.001).

**Table 2:** Multiple logistic regression parameter estimates and variable classes that predict incidence of FAW.

Parameter	Variable classes	DF	Estimate	Standard error	Wald 95% confidence limits		Chi-Square	Pr > Chi Square	Odds ratio
Intercept		1	-4.120	0.268	-4.644	-3.595	236.78	<.0001	0.02
Cropping Season	Irrigation	1	1.458	0.131	1.200	1.715	123.19	<.0001	4.30
	Main	0	0.000	0.000	0.000	0.000	.	.	1.00
Previous crop	Potato	1	0.686	0.185	0.323	1.048	13.73	0.0002	1.98
	Finger millet	1	0.949	0.187	0.581	1.316	25.62	<.0001	2.58
	Wheat	1	0.786	0.182	0.429	1.143	18.61	<.0001	2.20
	Maize	1	0.909	0.197	0.524	1.294	21.41	<.0001	2.48
	Snow pea	1	1.720	0.229	1.272	2.169	56.49	<.0001	5.58
	barley	1	0.597	0.219	0.168	1.026	7.45	0.0063	1.82
	Fallow	1	0.265	0.215	-0.157	0.686	1.52	0.2181	1.30
	Nug	1	0.672	0.233	0.216	1.129	8.33	0.0039	1.96
	Tomato	1	1.019	0.272	0.485	1.552	14.01	0.0002	2.77
	Onion	0	0.000	0.000	0.000	0.000	.	.	1.00
Weed abundance	Low	1	0.276	0.064	0.151	0.401	18.710	<.0001	1.32
	Medium	1	0.036	0.052	-0.066	0.138	0.470	0.4924	1.04
	High	0	0.000	0.000	0.000	0.000	.	.	1.00
Growth stage	Seedling	1	0.153	0.085	-0.014	0.321	3.230	0.0722	1.17
	Early whorl	1	0.118	0.051	0.018	0.218	5.320	0.0211	1.13
	Late whorl	0	0.000	0.000	0.000	0.000	.	.	1.00
Scale		0	1	0	1	1			2.72

DF, degrees of freedom.

Fields previously cropped with wheat (*Triticum aestivum*), barley (*Hordeum vulgare*), nug (*Guizotia abyssinica*), or potato (*Solanum tuberosum*) also showed significantly increased likelihood of FAW incidence (**Table 2**). Fallow plots, however, had no significant effect on FAW risk (OR = 1.30, p = 0.218), indicating limited pest carryover during non-host periods (**Table 2**). Weed abundance and maize growth stage were also identified as significant predictor variables for FAW incidence. Fields with low weed abundance showed a moderately higher likelihood of FAW infestation (*OR* = 1.32, *p* < 0.001), whereas fields with less frequent weeding tended to experience lower infestation rates. On the other hand, maize plants at the early whorl growth stage were significantly more susceptible to FAW attack, showing a slightly higher likelihood of infestation (*OR* = 1.13, *p* < 0.02; **Table 2**).

Analysis of parameter estimates for LDP (**Table 3**) revealed consistent seasonal trends, with irrigated maize fields showing an approximately threefold higher likelihood of severe leaf damage compared to fields cultivated during the main cropping season (*OR* = 2.9, *p* < 0.0001). Because the previous crop was a weak predictor of LDP overall, fields preceded by crops other than snow pea (*Pisum sativum*), nug (*Guizotia abyssinica*), or tomato (*Solanum lycopersicum*) did not show a significant effect on LDP. However, fields preceded by snow pea (*P. sativum*), nug (*G. abyssinica*), or tomato (*S. lycopersicum*) exhibited an approximately 40% lower likelihood of severe leaf damage compared with fields previously planted with onion (*Allium cepa*). Growth stage of maize, and stages of larval instars were also identified as significant predictors of LDP. Maize plants at the seedling stage exhibited a higher likelihood of severe leaf damage (OR = 0.06, *p* < 0.0001) compared with seedlings at the late whorl stage. Similarly, plants at the early whorl stage showed a significantly lower likelihood of LDP (OR = 0.09, *p* < 0.001) than those at the late whorl stage. Early larval stages (first and second instar) were associated with a greater likelihood of increased LDP than the sixth-instar larvae (OR = 1.4, *p* = 0.003).

**Table 3:** Multiple logistic regression parameter estimates of variables that predict FAW LDP.

Parameter	Variable classes	DF	Estimate	Standard error	Wald 95% confidence limits		Chi-Square	Pr > ChiSquare	Odds ratio
Intercept		1	-0.6218	0.1771	-0.9689	-0.2747	12.33	0.0004	0.5
Season	Irrigation	1	1.0585	0.0626	0.9359	1.1812	286.1	<.0001	2.9
	Main	0	0.0000	0.0000	0.0000	0.0000	.	.	1.0
Previous crop	Potato	1	-0.2446	0.1319	-0.5031	0.0139	3.44	0.0637	0.8
	Finger millet	1	0.1209	0.1329	-0.1396	0.3814	0.83	0.3629	1.1
	Wheat	1	-0.1429	0.1257	-0.3893	0.1035	1.29	0.2556	0.9
	Maize	1	-0.0353	0.1423	-0.3142	0.2435	0.06	0.8038	1.0
	Snow pea	1	-0.4395	0.1665	-0.7659	-0.1131	6.97	0.0083	0.6
	barley	1	-0.2001	0.1624	-0.5184	0.1181	1.52	0.2178	0.8
	Fallow	1	-0.0172	0.1490	-0.3093	0.2749	0.01	0.9081	1.0
	Nug	1	-0.5389	0.1856	-0.9026	-0.1751	8.43	0.0037	0.6
	Tomato	1	-0.5366	0.2642	-1.0544	-0.0188	4.12	0.0423	0.6
	Onion	0	0.0000	0.0000	0.0000	0.0000	.	.	1.0
Growth stage	Seedling	1	-0.4550	0.0750	-0.6019	-0.3081	36.84	<.0001	0.6
	Early whorl	1	-0.1435	0.0430	-0.2278	-0.0592	11.14	0.0008	0.9
	Late whorl	0	0.0000	0.0000	0.0000	0.0000	.	.	1.0
Larval instar	1st-2nd instar	1	0.3182	0.1099	0.1028	0.5336	8.38	0.0038	1.4
	3rd-5th instar	1	0.0789	0.0445	-0.0083	0.1661	3.15	0.0760	1.1
	6th instar	0	0.0000	0.0000	0.0000	0.0000	.	.	1.0
Scale		0	1.0000	0.0000	1.0000	1.0000			2.7

**Figure 3:**
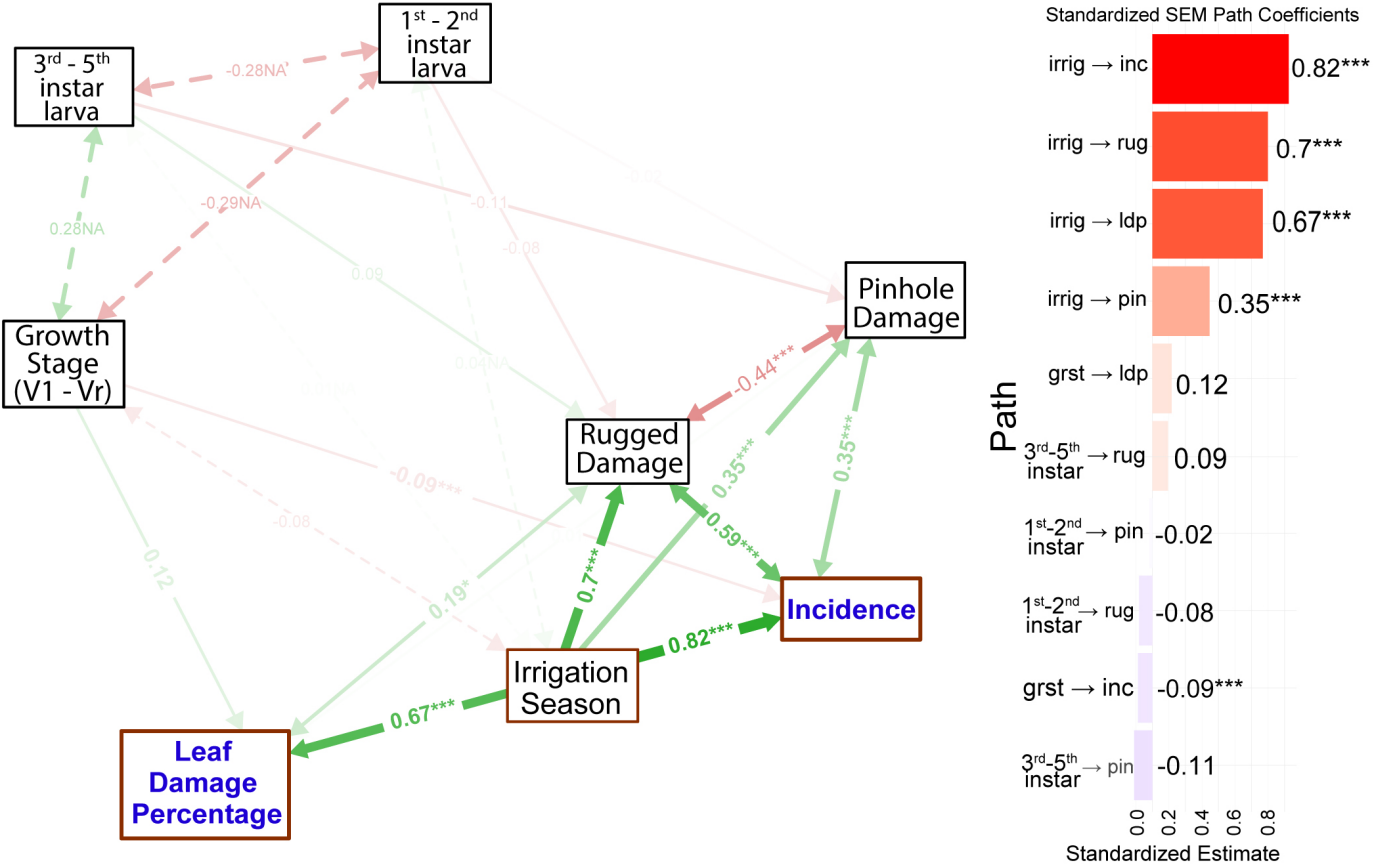
Structural equation model illustrating the causal relationships among factors contributing to FAW incidence and leaf damage. Irrig, irrigation; inc, incidence; rug, rugged damage; ldp, leaf damage percentage; pin, pinhole damage. Green arrows indicate positive associations, whereas red arrows indicate negative associations. Arrow thickness reflects the magnitude of the path coefficients. *, and *** denote significance at p < 0.05 and 0.001, respectively. Broken arrows and NA indicate non-significant relationships.

**Figure 4:**
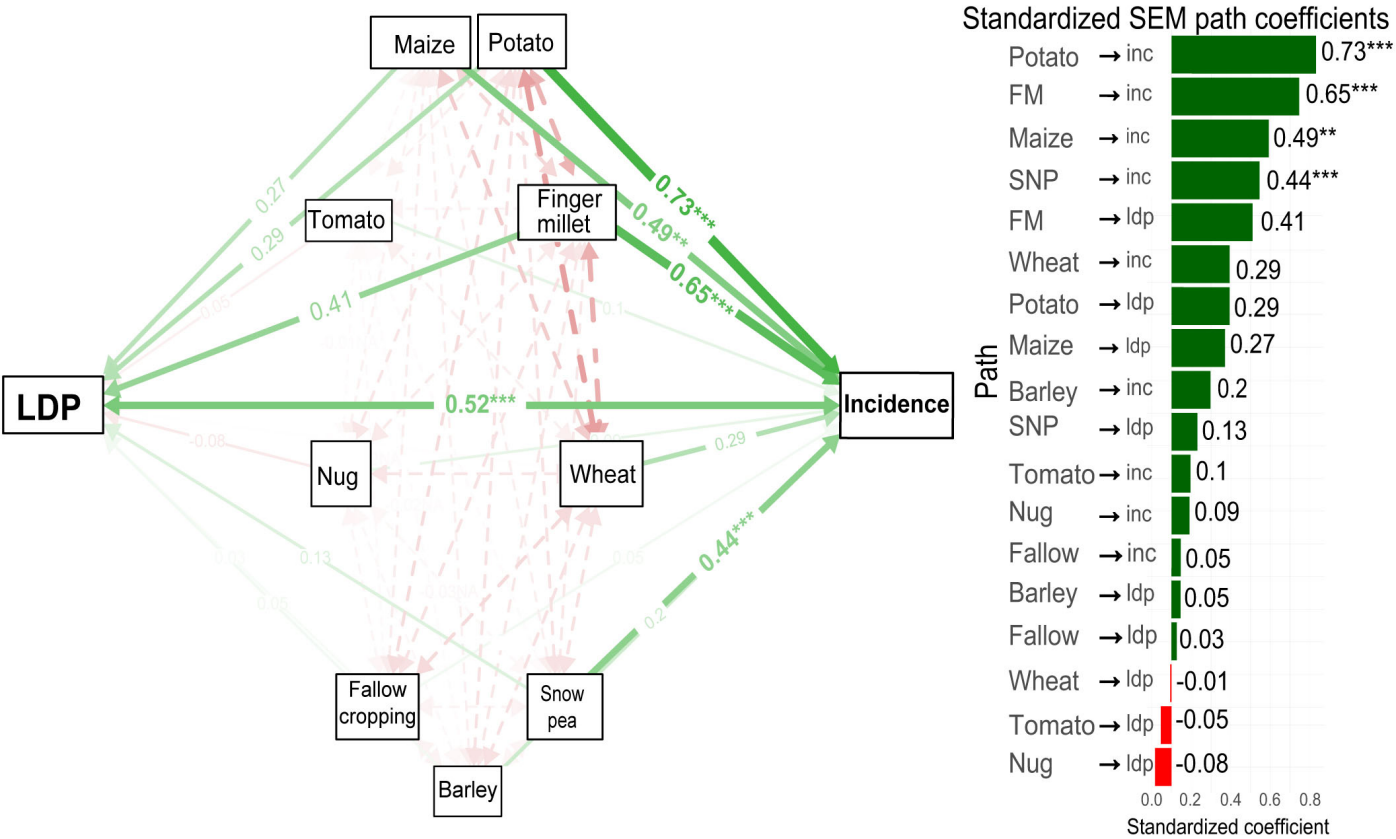
Structural equation model (SEM) illustrating the relationships among previous crops and FAW incidence and leaf damage percentage. FM, finger millet; SNP, snow pea; inc, incidence; ldp, leaf damage percentage; pin, pinhole damage. Green arrows indicate positive associations, whereas red arrows indicate negative associations. Arrow thickness reflects the magnitude of the path coefficients. **, and *** denote significance at p < 0.01 and 0.001, respectively. Broken arrows indicate non-significant relationships.

The structural equation model (**Figure 3**) revealed that irrigation-season maize cropping was significantly and strongly associated with higher FAW incidence (path coefficient = 0.82, *p* < 0.001) and with increased LDP (LDP; path coefficient = 0.67, *p* < 0.001). Irrigation-induced damage was also significantly correlated with both ragged damage (path coefficient = 0.70, *p* < 0.001) and pinhole damage. On the other hand, the growth stage of the crop showed a negative association with FAW incidence (**Figure 3**). The SEM analysis on the relationships of previous crops with incidence and severity showed that Potato had the strongest positive effect on FAW incidence (β = 0.73, *p* < 0.001) followed by finger millet (β = 0.65, *p* < 0.001), and maize (β = 0.49, *p* < 0.001) and snow pea (β = 0.44, p < 0.001) (**Figure 4**). Potato and finger millet rotations were associated with substantially higher infestation than non-rotated maize. Despite strong association of previous crops with FAW incidence, LDP exhibited moderate patterns with a non-significant association with leaf damage percentage.

### FAW larvae exhibited minimal host discrimination under controlled cage condition

Host preference assays using larvae from two adult females under controlled cage conditions showed no host discrimination among maize, barley, and finger millet, all of which experienced complete destruction within 30 DAH (*p* > 0.05) (**Supplementary File 1**: **Supplementary Table S1**; **Supplementary Figures S3A, B**). In contrast, wheat exhibited moderate resistance and significantly lower damage than finger millet, barley, and maize (*p* < 0.001), while tef sustained minimal damage at 30 DAH (**Supplementary File 1**: **Supplementary Figures S3C, D**; **Supplementary Table S1**). Estimated marginal means confirmed these trends: finger millet and barley reached 100% damage, maize reached 98.3%, wheat showed 75% damage, and tef experienced the least damage (10%) when co-cultivated with maize throughout the 30-day observation period (**Supplementary File 1**: **Supplementary Table S2**).

**Figure 5:**
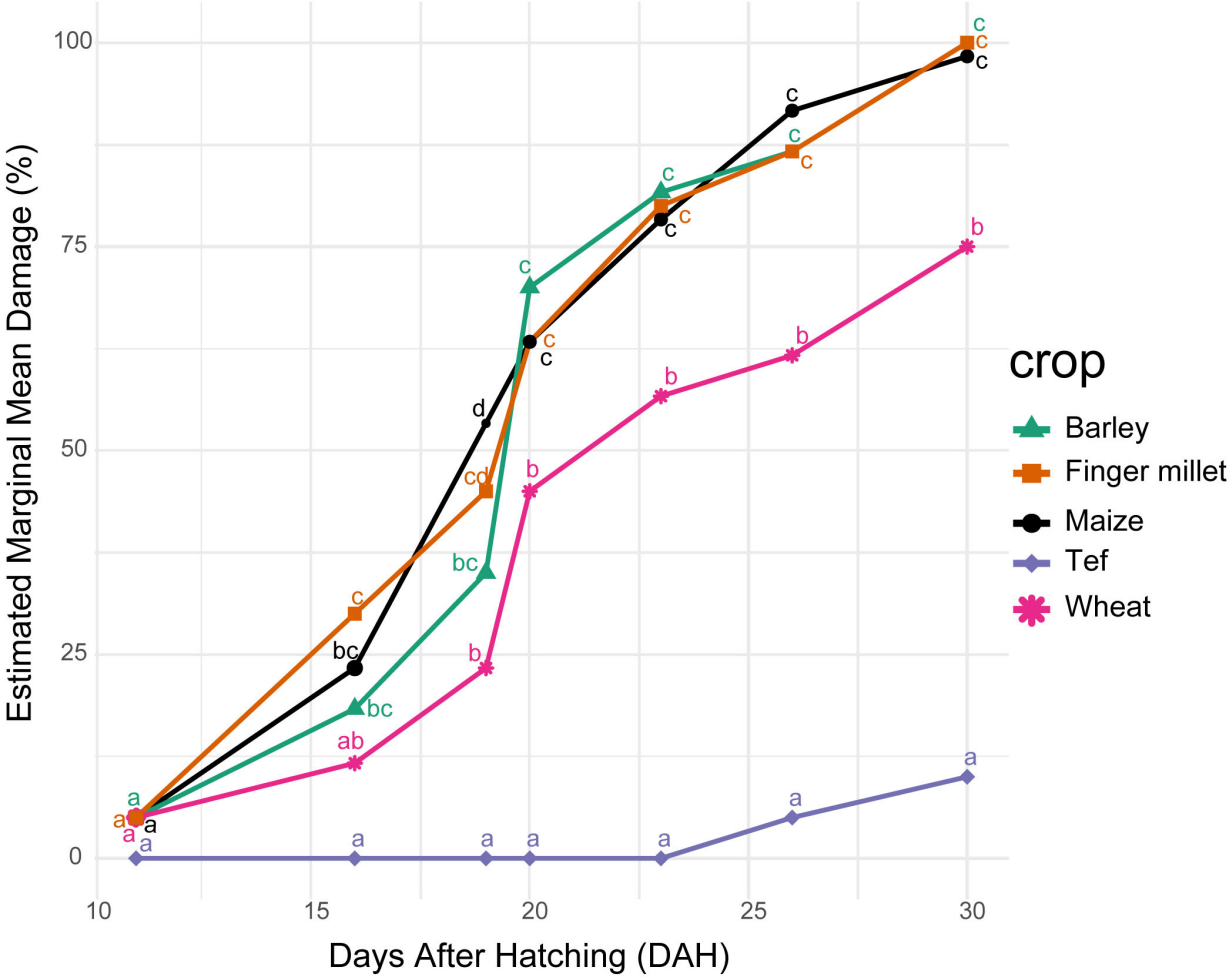
Estimated marginal means of leaf damage (%) across days after hatching (DAH) of *Spodoptera frugiperda* larvae that emerged from two female moths over a 30-day observation period on cereals co-cultivated with maize.

Leaf damage progressed gradually over time, with the first noticeable symptoms appearing at 11 DAH. At this stage, damage across all cereals was minimal and not statistically different (*p* > 0.05; **Figure 5**; **Supplementary File 1**: **Supplementary Table S3**), consisting mainly of light leaf scratching, followed by a slight increase up to 15 DAH (**Figure 4**). Damage progression in finger millet, maize, and barley did not differ significantly until 16 DAH. In contrast, wheat exhibited significantly lower damage than finger millet, and no damage was recorded on tef at this time point. A sharp increase in damage occurred at 19–20 DAH, during which barley, finger millet, and maize showed statistically similar and substantially higher damage compared with wheat. Tef remained the least affected, with no measurable damage until 23 DAH (**Figure 5**). The damage progressively continued and at 30 DAH, complete plant damage was observed on barley, finger millet, and maize. Wheat showed significantly lower overall damage compared with these cereals, although its leaf tissue was fully consumed and surviving panicles contributed to a moderate damage estimate (Emmean = 75%; **Figure 5**; **Supplementary File 1**: **Supplementary Table S2**). Tef exhibited significantly lower damage than all other cereals when co-cultivated with maize. However, when cereals were co-cultivated in the absence of maize, complete damage occurred across all, including tef, finger millet, wheat, and barley, within 30 days after larval hatching (DAH) (**Supplementary File 1**: **Supplementary Figures S4**, **S5**).

## Discussion

A significantly higher incidence of FAW during the Ethiopian irrigation season (typically November–April) compared to the main rain-fed cropping season (June–October) is attributed to multiple interacting factors. The irrigation season in tropical regions like Ethiopia, and basically areas such as the Nile River basin, are generally characterized by warmer temperatures, which provide optimal temperature and humidity conditions for FAW reproduction (Wu et al., 2022; Adan et al., 2024). Moreover, the continuous availability of irrigated maize during this period coincides with the pest’s peak reproductive phase, contributing to a significant increase in infestation and severity relative to rain-fed fields (Kartakis et al., 2025). The higher incidence could also be attributed to the smaller maize cultivation area compared to the main season. During the main season, infestations are dispersed across extensive maize fields, reducing local severity, whereas in the irrigation season, FAW populations concentrate within the limited maize plots at KIS, thereby increasing infestation incidence and severity. This suggests that irrigated maize acts as a survival spot during the off-season. Such continuity in host availability facilitates ongoing population buildup and enhances habitat suitability for FAW, aligning with global projections of its potential persistence in tropical and subtropical agroecosystems (Abdel-Rahman et al., 2023; Amaro et al., 2025). The irrigation season also exhibited a higher percentage of leaf damage than the main cropping season, with rugged feeding type being the dominant type of injury. This suggests a higher abundance of late-instar larvae (5th and 6th instars) during the irrigation season compared to the main season, implying that more larvae are likely to develop into reproductive adults during this period. Hence, effective FAW management during the irrigation season could play a critical role in reducing pest buildup and subsequent infestation pressure (Yang et al., 2021).

The host preference tests showed that the progeny from only two female adults were capable of causing substantial damage, a destruction of almost all the crops under investigation. The cage conditions, however, provided a protected environment which excluded natural enemies, and likely contributed to elevated larval densities and may therefore have amplified the observed levels of damage compared to the natural field situations. Nonetheless, the consistently lower damage in wheat compared to maize, finger millet, and barley supports the interpretation that host traits, such as the stronger panicle structure of wheat, contributes to its greater tolerance. However, the low damage observed on tef when grown alongside maize may not indicate inherent resistance, as the severe injuries recorded in the absence of maize confirms that tef can also be regarded as an alternative host. Moreover, these results were consistent with the highly polyphagous feeding behavior of FAW (Montezano et al., 2018; Sotelo-Cardona et al., 2021; Lu et al., 2023). Thus, while the experimental set-up may have intensified feeding pressure, the relative differences among host species remain informative.

The crop grown before maize in the preceding season significantly influenced FAW incidence and severity, highlighting crop rotation as a key driver of FAW dynamics. Higher incidence under monocropping is consistent with previous studies showing that continuous cropping favors persistence of *Spodoptera frugiperda* by providing a continuous supply of suitable hosts (Jalloh et al., 2023; Soujanya et al., 2024; Srinivasa Rao et al., 2025). In contrast, fallow periods were associated with a reduced risk of infestation, likely by limiting pest carryover between consecutive cropping seasons. Onion rotations exerted a suppressive effect on FAW incidence in the following season; However, these same fields exhibited higher LDP than those rotated with snow pea or tomato. This pattern is consistent with reports of FAW infestations in onion fields ranging from 5% to 20% (Soumia et al., 2023). Such contrasting effects suggest that factors driving initial infestation differ from those influencing subsequent feeding severity, as early infestation levels do not reliably predict larval damage (Sotelo-Cardona et al., 2021). Conversely, snow pea rotations were associated with a higher incidence but with approximately 40% lower severity, suggesting that residues or microhabitats left by snow pea may favor early adult colonization while simultaneously limiting larval feeding once maize is established. This highlights the differential effects of rotation crops, potentially mediated through soil microbial legacy (Wang et al., 2021).

Although crop rotation is generally expected to reduce pest pressure, it appeared to have limited impact on lowering FAW incidence. In some cases, rotation may even increase pest pressure, as rotations involving potato and finger millet resulted in higher FAW incidences than continuous maize cultivation (Tajdar et al., 2025). While maize is the primary host of *Spodoptera frugiperda*, host preference is highly plastic (Montezano et al., 2018) and is influenced by plant nutritional quality, developmental stage, and physicochemical traits (Ortiz-Carreón et al., 2025; Vasquez et al., 2024). Under certain conditions, alternative hosts such as potato and finger millet may provide more favorable succulent tissues and feeding substrates than maize at comparable growth stages, resulting in increased larval feeding activity and greater damage severity. Despite the higher silica deposition and greater tissue toughness observed in finger millet relative to maize (Jadhao et al., 2020), its smaller plant architecture and less protected meristematic tissues may increase susceptibility to complete destruction under intense feeding pressure by *S. frugiperda*, while supporting larval survival for longer period. In contrast, maize plants possess thicker stems and more robust structural features, allowing partial survival in later growth stages. Similarly, potato plants may suffer more extensive defoliation than maize due to their softer and more digestible leaf tissues, which may facilitate FAW survival during off-season periods. Controlled cage experiments also showed that FAW was capable of causing severe damage, with losses reaching 100%, indicating that the absence of maize alone did not prevent attack. The higher incidence of FAW following crop rotations, together with the lack of a strong host preference, indicates that the pest can persist during the irrigation season regardless of the crop grown (Lu et al., 2023). This suggests that the “green bridge” effect is not exclusively dependent on maize, as multiple alternative hosts are capable of sustaining FAW populations, in some cases even more effectively than maize. Off-season irrigation, therefore, provides suitable resources for survival during normally unfavorable tropical periods, underscoring the need for rigorous management interventions to prevent severe infestations in subsequent seasons.

Field vegetation structure further modulated infestation patterns. The moderately higher likelihood of FAW infestation in fields with low weed abundance suggests that weeds can disrupt host-location behavior. Because FAW moths strongly prefer maize for oviposition (Sotelo-Cardona et al., 2021), surrounding weeds may mask or modify volatile cues from maize seedlings (Wang et al., 2023), or act as physical barriers that reduce egg laying, especially when crop phenology influences oviposition decisions (Cheruiyot et al., 2021; Xiao et al., 2025).

These findings give a clue that FAW persistence might not be constrained by host availability alone, but it might be reinforced by field-level processes operating throughout the rotation cycle. Such effects may arise from crop-specific soil conditioning, including changes in physicochemical properties (Jalloh et al., 2023) or from shifts in soil microbiome structure and function that influence plant resistance to herbivores (Howard et al., 2020a, Howard et al., 2020b, Wang et al., 2021). Thus, designing rotation or intercropping schemes that enhance soil health, rather than simply alternating hosts, might be investigated in future studies to suppress both infestation and feeding damage.

Together, these results indicate that off-season irrigation creates conditions that maintain FAW populations across seasons regardless of crop type, reinforcing the need for management strategies that operate beyond the crop choice. These findings emphasize a combination of measures will be required to reduce pest carry-over and prevent population build-up before maize is replanted. Integrating pest management strategies into a coordinated, landscape-level framework would be essential for lowering FAW pressure in subsequent seasons and improving the long-term resilience of cereal cropping systems.

## Conclusion

The rapid global expansion of fall armyworm (FAW), combined with its polyphagous nature and exceptionally broad host range, underscores its emergence as one of the most significant insect pests of the present era. This expansion inevitably drives increased reliance on chemical insecticides, which not only heighten environmental risks but also accelerate the pest’s adaptation to multiple control agents, including transgenic crops. Our findings highlight the critical role of off-season irrigated non-maize hosts as a “green bridge” contributing significantly to maintaining FAW populations during periods that would otherwise limit its reproduction. Breaking or minimizing this green-bridge effect, through intensified off-season management strategies, should therefore be considered a core component of climate-resilient and integrated pest management (IPM) strategies aimed at reducing seasonal carryover. Future research should prioritize management strategies beyond crop choice or rendering the calibrated use of crop rotations informed by host-derived chemical cues influencing FAW behavior, shifts in soil physicochemical properties, and soil microbiome dynamics, providing a more mechanistic understanding of pest–environment interactions and advancing sustainable FAW management frameworks.

## Data Availability

The original contributions presented in the study are included in the article/**Supplementary Material**. Further inquiries can be directed to the corresponding author.

## Supplementary material

Supplementary Table 1 pairwise comparison of leaf damage estimates

Supplementary Table 2 Estimated Marginal Means (EMMs) for FAW Damage Across Test Crops 30 DAH

Supplementary Table 3 Estimated Marginal Means (EMMs) for FAW Damage Across Test Crops 11 DAH

Supplementary Figure 1 Sample pictures from the field survey showing different levels of leaf damage intensities of FAW

Supplementary Figure 2 Controlled cage experiment and artificial infestation setup for fall armyworm

Supplementary Figure 3 Feeding damage caused by fall armyworm on cereals co-cultivated with maize

Supplementary Figure 4 Feeding damage caused by FAW larvae on co-cultivated cereal hosts in the absence of maize.

Supplementary File 1 .

Supplementary File 2 Leaf damage data of FAW on cereals when co-cultivated with maize

Supplementary File 3 Leaf damage data of FAW on cereals when co-cultivated without maize

Supplementary File 4 Pest survey data during the irrigation and main cropping seasons
