# Supplementary material for: Non-maize hosts outperform maize in sustaining fall armyworm population during off-season irrigation in the tropics
Source: Front Plant Sci. 2026 Feb 25;17:1768817. doi: 10.3389/fpls.2026.1768817 (PMC12975733; doi:10.3389/fpls.2026.1768817)
Supplement: Supplementary Figure 1 — Sample pictures from the field survey showing different levels of leaf damage intensities of FAW [file DataSheet1.docx]

**Non-maize hosts outperform maize in sustaining fall armyworm population during off-season irrigation in the tropics**

Yohannes Ebabuye Andargie^1,2,3^, Mintesnot Worku Bogale^4^, Abaynew Jemal Jenber^3^, Alemu Abate^3^, Dong-Sun Lee^5^, Jae-Ho Shin^1,2*^

^1^Department of Applied Biosciences, Kyungpook National University, Daegu, Republic of Korea

^2^NGS Core Facility, Kyungpook National University, Daegu, Republic of Korea

^3^Department of Plant Sciences, Bahir Dar University, Bahir Dar, Ethiopia

^4^Amhara Agricultural Research Institute, Bahir Dar, Ethiopia

^5^Bio-Health Materials Core-Facility Center, Jeju National University, Jeju, Republic of Korea

*Author for correspondence: Jae-Ho Shin

Tel: +82 53 950 5716

jhshin@knu.ac.kr

**Supplementary file 1**

Table S1. pairwise comparison of leaf damage estimates

| **Contrast** | **Estimate** | **SE** | **df** | **t.ratio** | **p.value** |
| --- | --- | --- | --- | --- | --- |
| Maize - Barley | 2.381 | 2.22 | 68 | 1.071 | 0.8205 |
| Maize - Finger millet | 0.476 | 2.22 | 68 | 0.214 | 0.9995 |
| Maize - Tef | 56.905 | 2.22 | 68 | 25.604 | <0.0001 |
| Maize - Wheat | 19.286 | 2.22 | 68 | 8.678 | <0.0001 |
| Bar - Finger millet | -1.905 | 2.22 | 68 | -0.857 | 0.9114 |
| Barley - Tef | 54.524 | 2.22 | 68 | 24.533 | <0.0001 |
| Barley - Wheat | 16.905 | 2.22 | 68 | 7.606 | <0.0001 |
| Finger millet - Tef | 56.429 | 2.22 | 68 | 25.39 | <0.0001 |
| Finger millet - Wheat | 18.81 | 2.22 | 68 | 8.463 | <0.0001 |
| Tef - Wheat | -37.619 | 2.22 | 68 | -16.927 | <0.0001 |

*SE = Standard error, df = degrees of freedom*

Table S2. Estimated Marginal Means (EMMs) for FAW Damage Across test crops 30 DAH

| **Crop** | **Emmean** | **SE** | **df** | **Lower CL** | **Upper CL** | **Group** |
| --- | --- | --- | --- | --- | --- | --- |
| Tef (*Eragrostis tef*) | 10 | 4.5 | 40.6 | –2.121 | 22.121 | a |
| Wheat (*Triticum aestivum*) | 75 | 4.5 | 40.6 | 62.879 | 87.121 | b |
| Maize (*Zea mays*) | 98.3 | 4.5 | 40.6 | 86.212 | 110.5 | c |
| Finger millet  (*Eleusine coracana*) | 100 | 4.5 | 40.6 | 87.879 | 112.121 | c |
| Barley (*Hordeum vulgare*) | 100 | 4.5 | 40.6 | 87.879 | 112.121 | c |

*Emmean = Estimated marginal means, SE = Standard error, df = degrees of freedom*

Table S3. Estimated Marginal Means (EMMs) for FAW Damage Across test crops 11 DAH

| **Crop** | **Emmean** | **SE** | **df** | **Lower CL** | **Upper CL** | **Group** |
| --- | --- | --- | --- | --- | --- | --- |
| Tef (*Eragrostis tef*) | 0 | 4.5 | 40.6 | –12.121 | 12.121 | a |
| Wheat (*Triticum aestivum*) | 5 | 4.5 | 40.6 | –7.121 | 17.121 | a |
| Maize (*Zea mays*) | 5 | 4.5 | 40.6 | –7.121 | 17.121 | a |
| Finger millet  (*Eleusine coracana*) | 5 | 4.5 | 40.6 | –7.121 | 17.121 | a |
| Barley (*Hordeum vulgare*) | 5 | 4.5 | 40.6 | –7.121 | 17.121 | a |

*Emmean = Estimated marginal means, SE = Standard error, df = degrees of freedom*


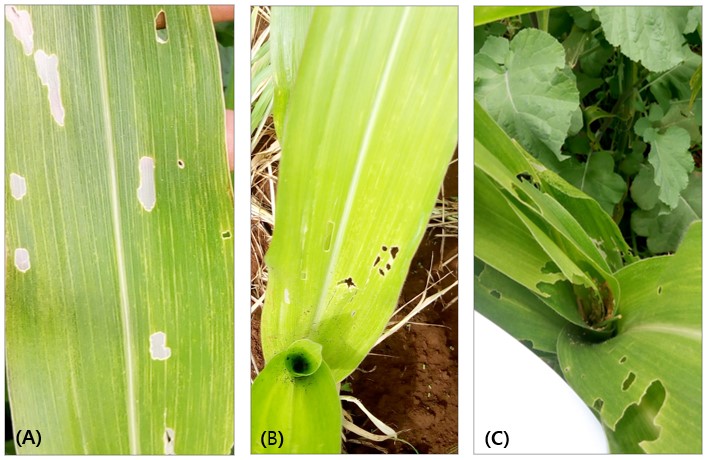
Figure S1. Sample pictures from the field survey showing different level of leaf damage intensities of FAW. (A) Scratching damage (B) Pinhole damage (C) Rugged damage


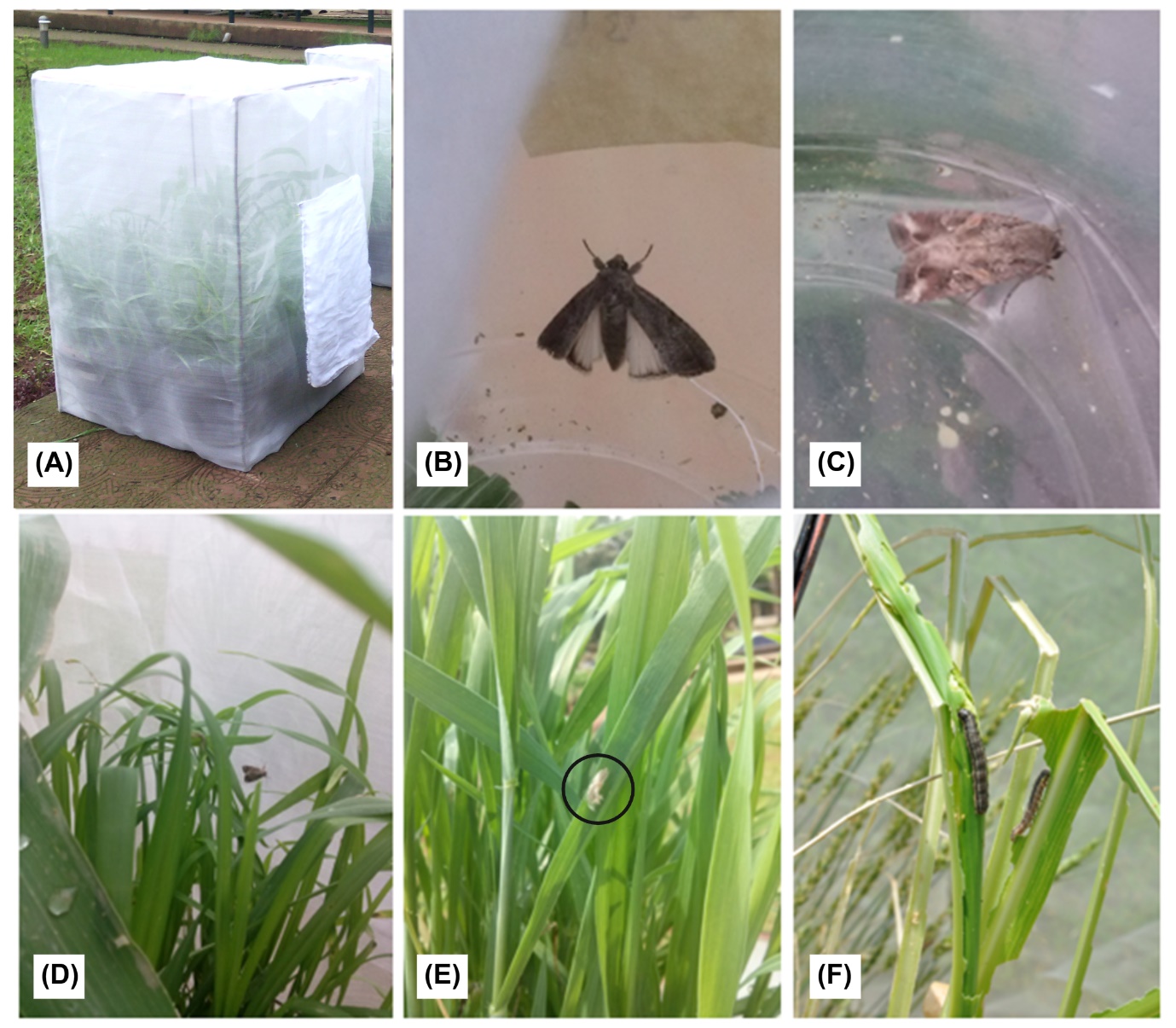
**Figure S2.** Controlled cage experiment and artificial infestation setup for fall armyworm. (A) Cage setup prepared for the release of two female and one male adult moths. (B) Female FAW adult prior to release. (C) Male FAW adult prior to release. (D) Male moth inside the cage after release. (E) FAW egg mass deposited on a barley leaf. (F) Characteristic larval feeding damage on maize leaves.


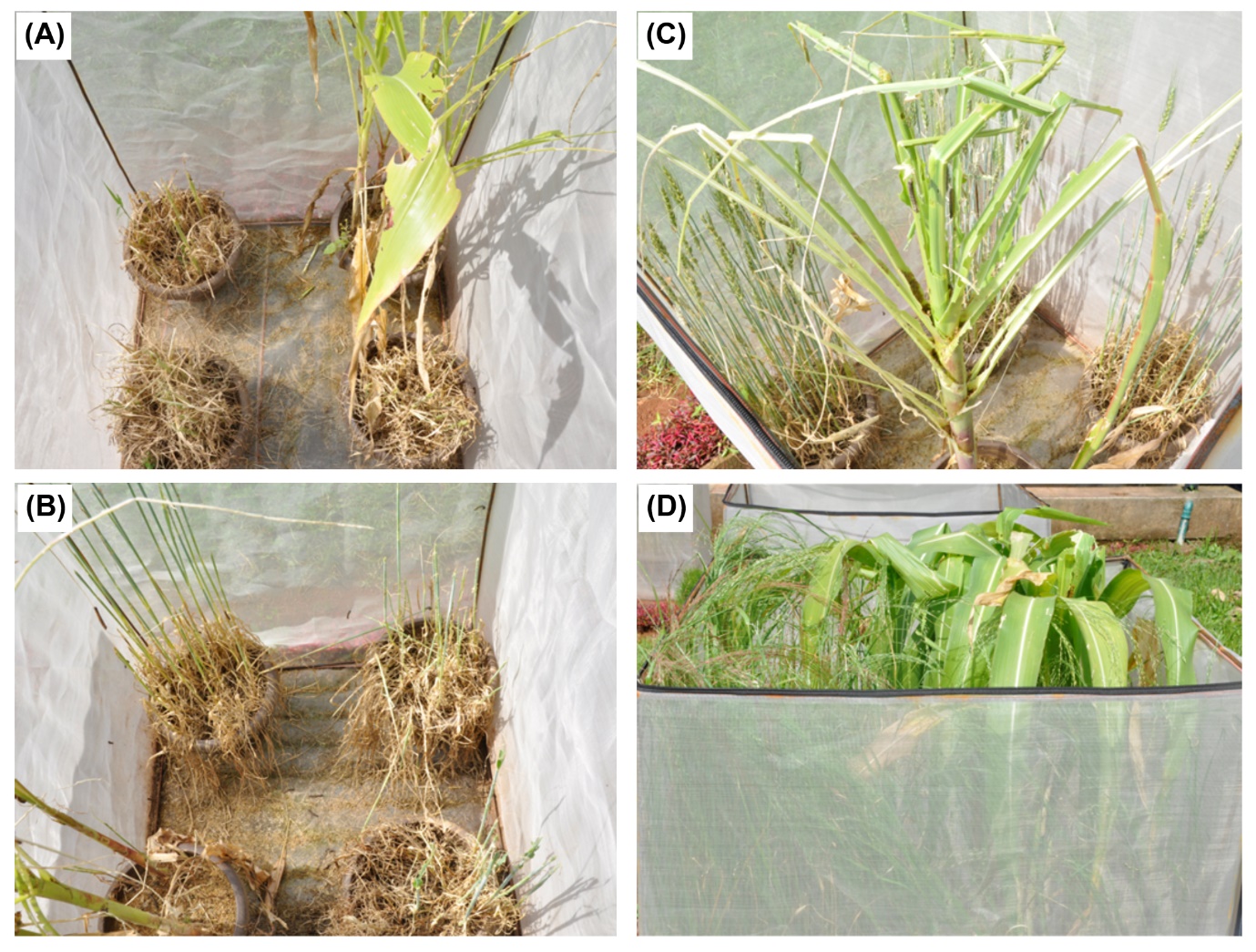


**Figure S3.** Feeding damage caused by fall armyworm on cereals co-cultivated with maize assessed under controlled cage conditions thirty days after larval hatching (DAH). (A) Finger millet with maize (B) Wheat with maize (C) Barley with maize (D) Tef with Maize at 30 DAH


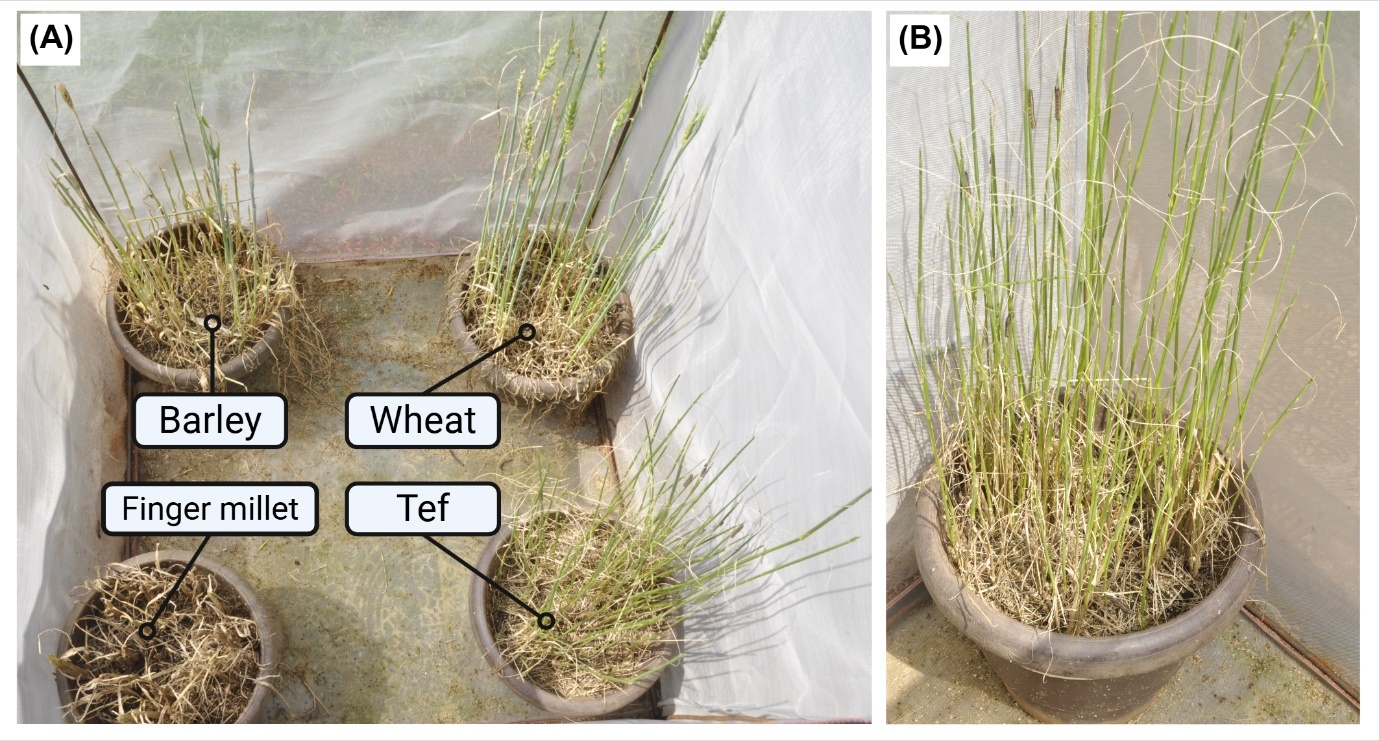


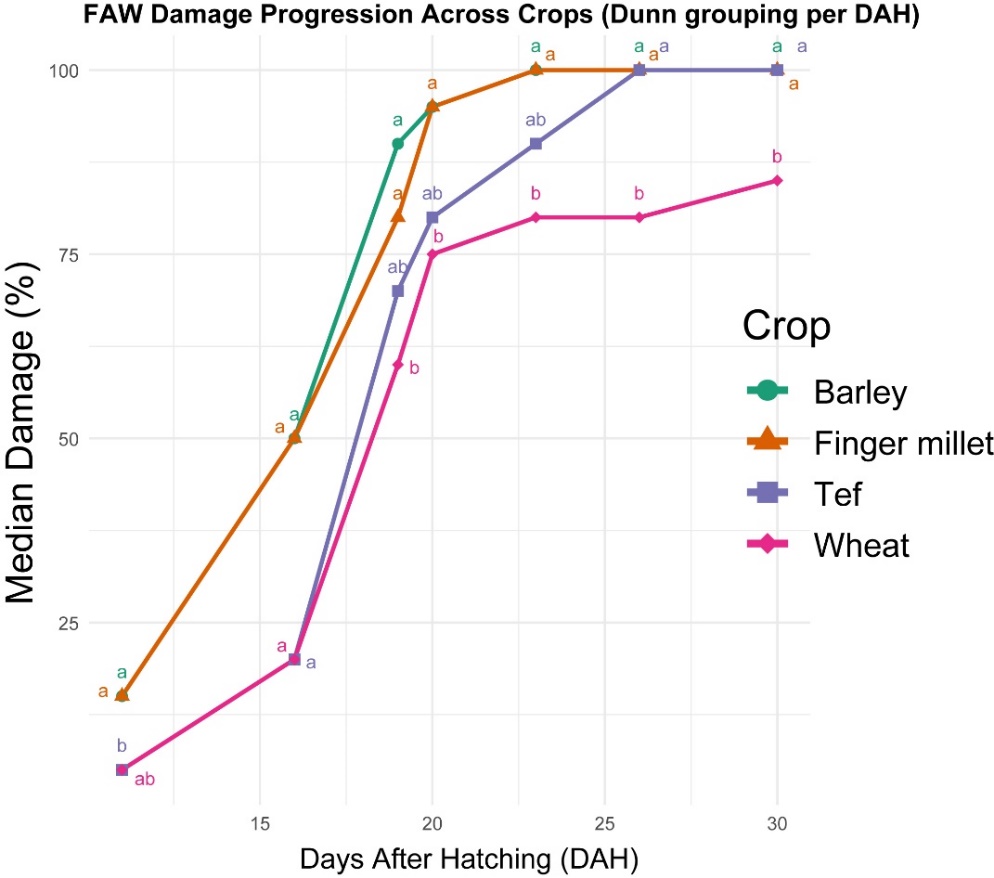
**Figure S4.** Feeding damage caused by FAW larvae on co-cultivated cereal hosts in the absence of maize. Damage levels were caused by larval progenies emerged from two female and one male adults, assessed under controlled cage conditions thirty days after larval hatching (DAH).

**Figure S5**. Estimated median feeding damage of FAW on co-cultivated cereals in the absence of maize under controlled cage conditions 30 days after larvae hatching (DAH).
